# Assessing the roles and challenges of librarians in dental systematic and scoping reviews

**DOI:** 10.5195/jmla.2021.1031

**Published:** 2021-01-01

**Authors:** Nena Schvaneveldt, Elizabeth M. Stellrecht

**Affiliations:** 1 nena.schvaneveldt@utah.edu, Assistant Librarian, Spencer S. Eccles Health Sciences Library, University of Utah, Salt Lake City, UT; 2 homann4@buffalo.edu, Senior Assistant Librarian, Clinical Librarian, and Liaison to the School of Dental Medicine, Health Sciences Library at Abbott, University at Buffalo, Buffalo, NY

## Abstract

**Objective:**

The objective of this study was to determine the scope of experience, roles, and challenges that librarians face in participating in dental and oral health systematic and scoping reviews to inform outreach efforts to researchers and identify areas for librarian professional development.

**Methods:**

The authors developed a twenty-three-item survey based on the findings of two recent articles about health sciences librarians' roles and challenges in conducting systematic and scoping reviews. The survey was distributed via electronic mailing lists to librarians who were likely to have participated in conducting dental systematic and scoping reviews.

**Results:**

While survey respondents reported participating in many dental reviews, they participated more commonly in systematic reviews than in scoping reviews. Also, they worked less commonly on dental and oral health reviews than on non-dental reviews. Librarian roles in dental reviews tended to follow traditional librarian roles: all respondents had participated in planning and information retrieval stages, whereas fewer respondents had participated in screening and assessing articles. The most frequently reported challenges involved the lead reviewer or review team rather than the librarians themselves, with time- and methodology-related challenges being most common.

**Conclusions:**

Although librarians might not be highly involved in dental and oral health systematic and scoping reviews, more librarian participation in these reviews, either as methodologists or information experts, may improve their reviews' overall quality.

## INTRODUCTION

Methodologies such as systematic and scoping reviews attempt to collect and synthesize all available evidence and involve evaluating studies for bias and combining them to provide recommendations for practice [1–3]. Researchers are attracted to the rigor and scientific approach of systematic-style reviews, and these syntheses can seem like a panacea to the ever-growing pool of health sciences literature. However, while these methodologies are useful for synthesizing available evidence for clinical practice, research focusing on these reviews has demonstrated that there is often a lack of reproducibility, calling into question the conclusions of previously published systematic and scoping reviews.

One possible solution to the issues of reproducibility and poor quality of reviews is the further involvement of librarians as expert searchers and methodologists. One study found that at least a third of the analyzed systematic reviews across all disciplines that had been indexed in February 2014 did not include a full report of a search strategy for at least one database, meaning these reviews were not reproducible [4], a finding that was also found in dentistry [5]. Further, a study of reviews indexed in 2017 found poor reporting, inconsistent methodology, and failure to adhere to best practices, such as searching for grey literature and in languages other than English [6]. An analysis of search strategies used in prosthodontics found that 95% of search strategies were not, in fact, systematic [7]. Indeed, errors such as not including all synonyms for a topic, not including subject headings, and not clearly reporting search strategies are widespread among reviews of all types [8]. This lack of recall is of particular concern in reviews: without a sensitive, reproducible search, the reader cannot be certain that all available evidence was gathered and synthesized.

Systematic and scoping reviews are on the rise in dental medicine as a whole. For instance, Saltaji and colleagues found that 1,188 systematic reviews were published in dental medicine between 1991 to 2012 [9], while a PubMed search of “systematic review”[ti] conducted in May 2020 limited to dental journals found more than 2,500 reviews published in the last 5 years alone. Similarly, a cursory search of PubMed with the term “scoping review”[tiab] limited to dental journals resulted in 91 articles, with 86 of the results published in the last 5 years. The explosion in popularity of scoping reviews may be due to the ability of these reviews to be conducted in a shorter time frame, lower cost, lack of necessary ethical clearance, desirability with journals, and number of citations they receive [10]. These factors may make these reviews attractive for those who are looking for a seemingly less involved methodology, such as junior researchers and faculty looking to give students exposure to research.

Beyond merely impacting the reviews themselves, systematic and scoping reviews utilizing poor methodology can also impact practice guidelines. One study found that periodontal guidelines were based on reviews with poor methodology, with errors such as excluding grey literature, relying only on keywords, and not reporting search strategies [11]. A lack of reporting in line with Preferred Reporting Items for Systematic Reviews and Meta-Analyses (PRISMA) was also found in restorative dentistry [12]. It is imperative to oral health that published systematic and scoping reviews are of the highest quality to create truly evidence-based practice guidelines.

It is important, therefore, that systematic and scoping reviews in oral health and dentistry employ rigorous methodology. While dentists and oral health experts have subject expertise, they might not have the expertise in review methodology or expert searching skills that are necessary for these review types. To fill this gap, librarians have taken on increasing roles in conducting systematic and scoping reviews, a number of which have been identified in a scoping review by Spencer and Eldredge [13].

As expert searchers, librarians are adept at developing thorough, sensitive literature searches, and their involvement improves the quality of systematic and scoping reviews [14]. Librarians can also serve as methodologists and ensure that the review type matches the review question, that the review question is answerable, and that the review methods are carried out well [15]. Librarians may be able to help subject experts determine which kind of review their questions would be suitable for and, thus, improve the quality of the final product [16]. However, librarians face barriers to assuming roles in systematic reviews other than searching and often want more time and training to assume these other roles [17]. Similarly, scoping reviews arose after systematic reviews and are far more ill defined, which has made identifying librarian roles for this type of review challenging [18]. However, as another form of knowledge synthesis, many similar issues apply.

Nicholson and colleagues report that librarians run into a number of challenges in conducting systematic and scoping reviews [19]. Many include similar challenges that affect the quality of systematic reviews overall, such as poorly formulated questions and a failure to follow proper methodology, which ultimately undermines the role and effort of the librarian in conducting quality searches. While these challenges have been reported for systematic and scoping reviews across the health sciences, some studies have focused on librarians' experiences in specific disciplines. For example, one study found that veterinary medical librarians were trained and often consulted on systematic reviews, but their participation in and demand for reviews was low [20].

A previous environmental scan of libraries and librarians supporting dental medicine in the United States and Canada found that 45% of libraries had an official systematic review service, whereas 86% of librarians indicated some level of participation in systematic reviews [21]. This same study found that 93% of accredited dental program had at least 1 librarian who served the college of dentistry, although some librarians performed a myriad of duties or shared liaison areas.

The present study investigates librarians' experience with performing systematic and scoping reviews in oral health and dentistry to identify unique challenges in these specialties. The results can inform librarians who support oral health and dentistry about potential challenges and opportunities in conducting these types of reviews and highlight further needs for research, training, or outreach. The authors seek to answer the following research questions: What are the roles of librarians and information specialists who conduct systematic or scoping reviews in oral health and dentistry? How do these roles and challenges compare to the roles and challenges discovered in the previously published research literature?

## METHODS

We constructed a twenty-three-question survey (supplemental appendix), based on the roles reported by Spencer and Eldredge [13] and challenges reported by Nicholson and colleagues [19]. After reviewing the literature for frequently reported roles and challenges, we added and combined items for the survey to ensure it was logical and would answer the research questions. The survey consisted of mostly multiple-choice questions for respondents to indicate their roles and challenges in conducting reviews. Some questions about numbers of reviews used text entry. We also collected demographic data about respondents' geography, experience in librarianship, experience with dentistry and oral health, and participation in different kinds of reviews.

The survey was granted exemption from institutional review board (IRB) review by the University of Utah (IRB 00124426). The survey was created in REDCap and distributed to several electronic mailing lists used by librarians who conduct systematic and scoping reviews as well as dental librarians: MEDLIB-L, Dental and Systematic Reviews Caucuses of the Medical Library Association, Canada Health Libraries Association/Association des bibliothèques de la santé du Canada Oral Health Interest Group, American Dental Education Association Evidence Based Dentistry Special Interest Group, and ExpertSearching. This method was chosen instead of contacting individuals and associations because we assumed that these electronic mailing lists would include all librarians who participated in reviews, including those with whom we were not familiar.

The email invitation contained consent information as well as an invitation to forward the email to other eligible colleagues. Data collection occurred from January 17, 2020–January 31, 2020, with a reminder email sent out at the midway point on January 24. Based on previously gathered information about the number of dental programs in the United States and Canada and librarians' involvement in systematic reviews [21], we anticipated no more than sixty eligible responses.

We analyzed survey responses using descriptive statistics, including the frequency of responses to multiple-choice questions and ranges and medians for entered numerals.

## RESULTS

### Respondent demographics

The survey received 36 eligible responses out of a total of 40 responses; 4 respondents indicated that they had not participated in systematic or scoping reviews and, therefore, were excluded. All respondents were from North America or Western Europe, with the United States (n=18) and Canada (n=8) having the largest representation. Respondents had a wide range of longevity both in their overall careers and with dentistry. Over half (56%, n=20) of respondents had worked as a librarian for 11–20 years, regardless of specialty. Most had spent less time working with dentistry: 42% (n=15) had ≤5 years of experience with dentistry, and 39% (n=14) had 6–10 years of experience with dentistry. Most (81%, n=29) respondents worked in academic settings, whereas the others worked in hospitals, government agencies, research agencies, or a combination thereof.

### Review demographics

**Figure 1:**
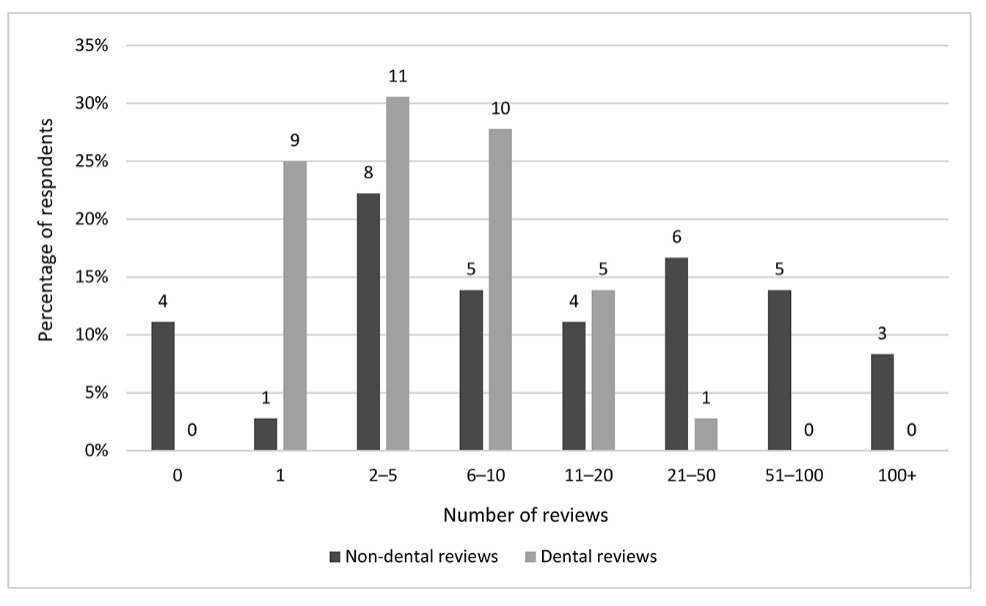
Numbers of dental and non-dental reviews conducted

We gathered information on the numbers of systematic and scoping reviews in which respondents participated to better contextualize their roles and challenges. There was a wide range (0–400) in number of non-dental reviews conducted by respondents: 4 respondents conducted no non-dental reviews, and 3 conducted ≥100 reviews (Figure 1). Most respondents (64%, n=23) had conducted 5–30 non-dental reviews, with a median of 15 reviews. Compared with non-dental reviews, respondents reported participating in fewer dental reviews. Thirty (83%) respondents reported participating in ≤10 dental reviews, although 1 respondent had conducted 40 dental reviews. Most respondents had conducted 1–17 dental reviews, with a median of 4 reviews.

**Figure 2:**
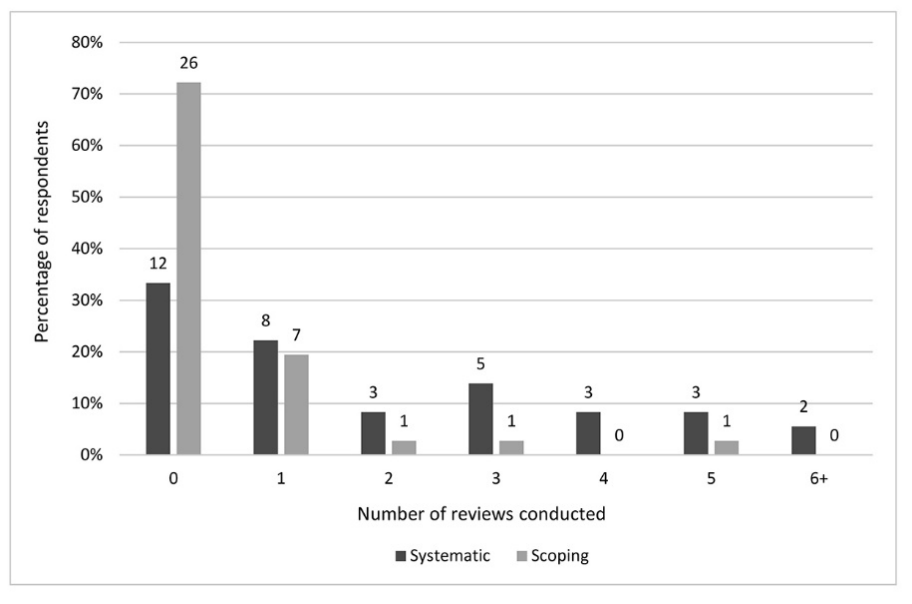
Number of published dental systematic and scoping reviews

Participating in dental systematic reviews that had resulted in publication was relatively uncommon: 94% (n=34) of respondents reported participating in ≤5 systematic reviews that were ultimately published or accepted for publication, and 33% (n=12) reported participating in no published systematic reviews. The median number of published dental systematic reviews was 1. Participating in dental scoping reviews that resulted in publication was even less common: the number of published scoping reviews ranged from 0–5; 74% (n=26) of respondents reported not having participated in a published scoping review (Figure 2).

**Figure 3:**
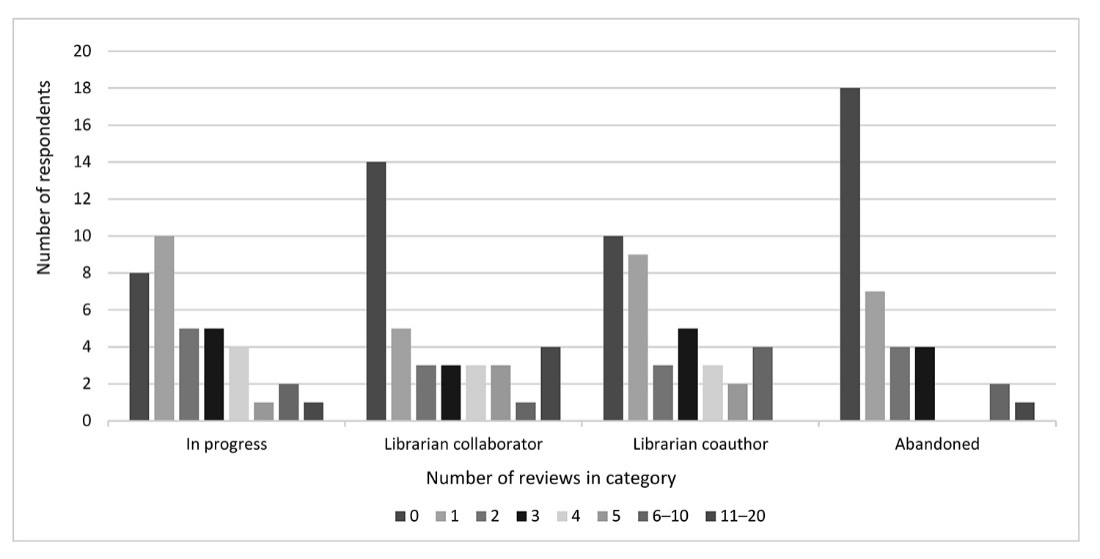
Numbers of reviews by category

We next asked respondents how many dental systematic and scoping reviews were in progress or had been collaborated on, coauthored, or abandoned (Figure 3). We defined collaboration as working longer-term with the review team; engaging in a wide array of duties including database selection, search strategy, and definition or refining of the topic; and possibly being acknowledged but not credited as a coauthor. We defined abandoned as either never finished or published.

### Librarian roles

**Figure 4:**
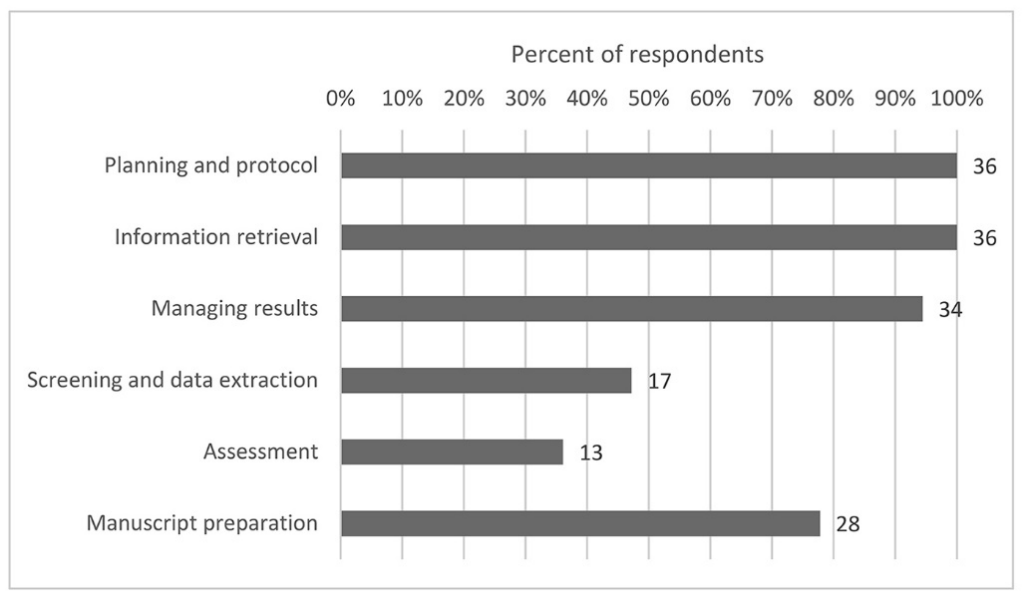
Dental librarian roles in different stages of the systematic or scoping review process

Respondents reported a range of involvement in different stages of the dental systematic or scoping review process (Figure 4).

All (100%, n=36) respondents were involved in the review planning stage. The most common roles were clarifying what was involved in a systematic or scoping review (89%, n=32), searching for previous and/or similar reviews (83%, n=30), formulating questions (64%, n=23), contributing to protocol development (61%, n=22), seeking guidance on selecting appropriate review methodology (50%, n=18), and locating and/or recommending journals that may publish the finished review (31%, n=11).

All (100%, n=36) respondents were also involved in the information retrieval stage. The most common roles were selecting the databases and information sources to search (100%, n=36), developing the search strategy (100%, n=36), searching grey literature (72%, n=26), and evaluating the search strategies (83%, n=30).

All (100%, n=36) respondents were involved with managing the results of information retrieval. The most common roles were de-duplicating search results (89%, n=32), documenting the search strategy (75%, n=27), consulting on and recommending the use of systematic review software (75%, n=27), and facilitating use of that software (47%, n=17).

Half (47%, n=17) of respondents were involved in the screening and data extraction stages. The most common roles were finding full texts of articles (47%, n=17), screening titles and abstracts (14%, n=5), screening full texts (6%, n=2), and performing data extraction (6%, n=2).

Only one-third (36%, n=13) of respondents were involved in the quality assessment stage. The most common roles were locating risk of bias tools (22%, n=8) and performing quality assessment (17%, n=6). No respondents reported performing risk of bias assessment.

Most (78%, n=28) respondents were involved in the manuscript preparation stage. The most common roles were writing the methods section (75%, n=27), editing the final manuscript (47%, n=17), being responsible for citation management in the manuscript (44%, n=16), and writing sections of the manuscript other than the methods section (11%, n=4).

### Librarian challenges

Finally, we asked a series of questions about challenges in three aspects of performing dental systematic and scoping reviews: the librarian's own ability and training, work with the lead reviewer, and work with the review team as a whole.

Most (69%, n=25) respondents reported challenges due to their own ability and training. The 2 top challenges were lack of subject knowledge (42%, n=15) and lack of time (42%, n=15). Almost a quarter (22%, n=8) of respondents indicated a lack of confidence in their ability, and 6% (n=2) reported inadequate training in conducting reviews. No respondents indicated a lack of administrative support for conducting reviews.

**Figure 5:**
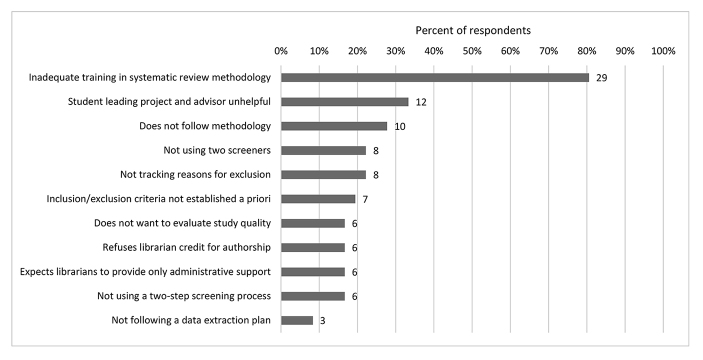
Challenges in working with the lead researcher

Most (83%, n=30) respondents also reported challenges in working with the lead reviewer during the review process (Figure 5). The most common challenges were the lead reviewer not having adequate experience or training in review methodology (81%, n=29), a mentor not being helpful in cases in which a student was leading the review (33%, n=12), and the lead reviewer not following proper review methodology (28%, n=10).

**Figure 6:**
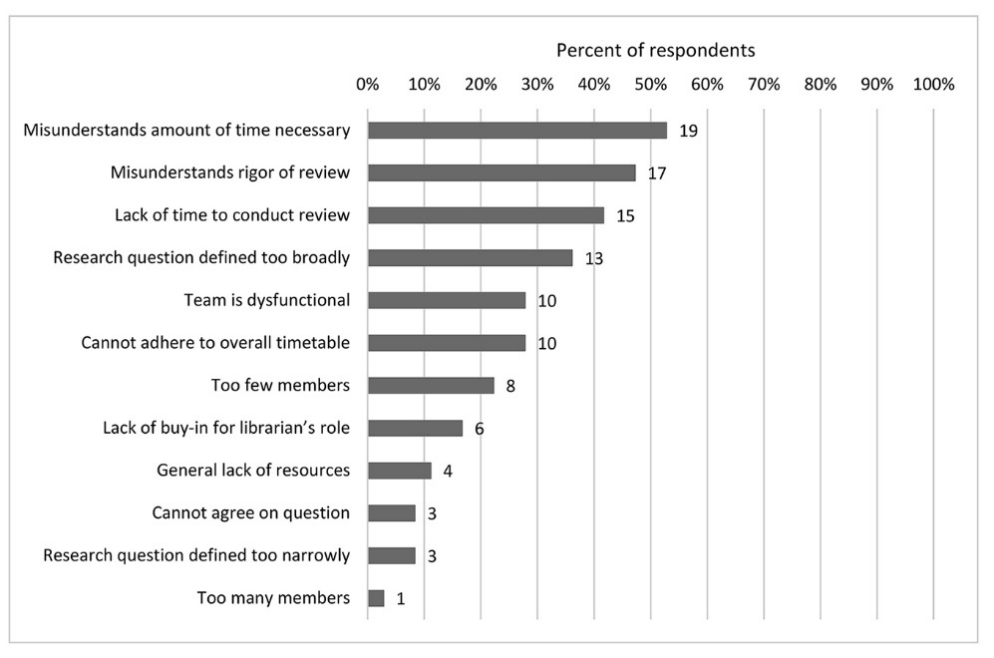
Challenges in working with the review team

Nearly three-quarters (72%, n=26) of respondents reported challenges in working with the entire review team (Figure 6). The most common challenge was that the team misunderstood the amount of time needed to conduct the review (53%, n=19). Relatedly, many respondents reported that the team lacked time to conduct the review (42%, n=15) and was unable to adhere to the review time table (28%, n=10). Challenges surrounding review methodology were also common: the review team often misunderstood the rigor involved (47%, n=17) or lacked the resources to conduct a review (11%, n=4). Challenges with the review question included the question being defined too broadly (36%, n=13) or too narrowly (8%, n=3) or the team not reaching agreement on the review question (8%, n=3). Furthermore, respondents reported interpersonal challenges: review teams were sometimes dysfunctional (28%, n=10), lacked buy-in from other team members about the librarian's role (17%, n=6), and were too small (22%, n=8) or too large (3%, n=1).

## DISCUSSION

This survey indicates that while librarians are involved in oral health and dental systematic and scoping reviews, their efforts may be just beginning to result in publications. While respondents reported being involved in one to forty dental reviews, almost all had fewer than five reviews published or accepted for publication at the time of the survey. One-third of respondents did not yet have any systematic or scoping reviews published. However, the number of dental reviews being published should likely increase over the next few years, as most respondents had one to four systematic or scoping reviews currently in progress. It is also encouraging that half of respondents had not had any reviews abandoned, whereas the other respondents had only between one to three reviews abandoned.

Although all librarians involved in dental systematic and/or scoping reviews were involved in information retrieval and management roles, many also played other roles. It was not surprising that the most frequently reported roles were selecting databases and resources to search as well as developing the search strategy, as the Cochrane Handbook for Systematic Reviews for Interventions discusses how creating search strategies can be very complex, and thus review teams can greatly benefit from having a librarian or information specialist as part of the team [3]. Although Spencer and Eldredge found little evidence of librarian involvement in question formulation, we found that over 60% of survey respondents reported having had a role in question formulation for dental reviews [3, 13]. Three-quarters of respondents coauthored at least 1 dental systematic or scoping review, which was reflected by the many respondents who reported writing the methodology section.

Of possible challenges experienced, respondents were less likely to report internal barriers (i.e., their own expertise and training) than external barriers (i.e., the lead reviewer and/or review team). The most frequently selected challenges related to review methodology or time. While there are a number of systematic review trainings available for librarians to learn review methodology, they are often general in focus and range from hour-long webinars to multiday trainings. Many respondents experienced issues where the lead researcher did not have adequate experience in methodology, or the team did not understand the rigor involved in the review methodology, echoing the findings of Nicholson and colleagues [19].

Indeed, there are few trainings about systematic reviews for oral health professionals, such as the “How to Conduct and Publish Systematic Reviews and Meta-Analyses” training from the American Dental Association [22]. However, more trainings for lead researchers, or more contact with knowledgeable librarians, could lead to a better understanding of the rigor of systematic review methodology. While Nicholson and colleagues found that librarians faced challenges concerning the review question, survey respondents involved in dental reviews experienced issues involving the team not understanding the amount of time needed to complete a review or not having the time to conduct the review. Ultimately, these challenges provide an opportunity for librarians to properly educate dental professionals about conducting systematic and scoping reviews so these challenges can be eliminated from the outset. Librarians can also serve as advocates for employing high-quality methodology, including protocol and question development, appropriate review selection, and reproducibility of literature searches.

Our results identified a possible lack of librarian experience with oral health and dentistry as a subject area, which might be why respondents reported less involvement in dental systematic or scoping reviews than reviews in other subject areas. Most survey respondents had worked as a librarian or information specialist for eleven to twenty years but spent half of this time or less supporting dentistry or oral health. This inexperience was also reflected in the median numbers of reviews in which respondents had participated, which was fifteen for non-dental reviews versus four for dental reviews. Consistently, the most frequently selected internal challenge experienced by librarians was a lack of subject knowledge in dentistry.

Currently, there are few known formal trainings for librarians to develop expertise in dentistry, most of which focus on teaching evidence-based dentistry. These resources include a LibGuide [23], professional conferences, and a training from the American Dental Association [24]. Also, the Medical Library Association Dental Caucus's meetings and electronic mailing list serve as ways for librarians to build expertise and network with colleagues and may be a group that is interested in developing continuing education around dental librarianship.

Another possible reason for lower librarian involvement in dental reviews is that there may be a lack of requests from dental and oral health professionals. This was cited as the largest barrier for librarian involvement in veterinary medicine [20], but it was important to note that the authors of that previous study stated that systematic and scoping reviews were still relatively uncommon in veterinary medicine, which appeared to be the opposite for dental reviews. Therefore, there may be opportunity for dental librarians to increase outreach to researchers who may be interested in conducting these types of reviews.

We anticipated obtaining a larger number of responses from North American librarians, because there were more than seventy accredited dental predoctoral programs in the United States and Canada, and a previous study indicated that most of these programs had a librarian who participated in systematic reviews [21]. However, many librarians who work with dentistry may share other liaison areas or have moved away from systematic or scoping reviews, limiting our response rate.

Another limitation of this study was that there was not a question specifically regarding consulting on reviews, which might account for why nearly half of respondents reported not having collaborated on any reviews but indicated that they had indeed been involved in dental reviews. It is also important to note that dental systematic and scoping reviews make up a small percentage of published reviews across all health sciences disciplines, which may account for why there seems to be lower librarian involvement in dental reviews.

## CONCLUSIONS

Oral health and dentistry systematic and scoping reviews are on the rise in popularity, and previous research demonstrates a need for improvements in their methodology and reporting, which can be attained through librarian involvement. Our results indicate that dental researchers may be unaware of the rigor and time involved in conducting systematic and scoping reviews. However, librarians are poised to educate them in the requisite methodology. More experienced dental librarians can build courses or tool kits to help those with less experience build their subject knowledge, which may remove barriers to collaborating on or coauthoring reviews. As dental practitioners depend on the recommendations made by systematic and scoping reviews to make evidence-based decisions for patient care, the quality of these reviews is paramount.

## Data Availability

Data associated with this article are available in openICPSR at https://www.openicpsr.org/openicpsr/project/119644/version/V2/view/.

## SUPPLEMENTAL FILE

**Appendix:** Survey instrument
