# Supplementary material for: Assessing the roles and challenges of librarians in dental systematic and scoping reviews
Source: J Med Libr Assoc. 2021 Jan 1;109(1):52–61. doi: 10.5195/jmla.2021.1031 (PMC7772970; doi:10.5195/jmla.2021.1031)
Supplement: Supplementary file 1 — Appendix: Survey instrument [file jmla-109-1-52-s01.pdf]

JMLA Journal of the Medical Library Association

SC
Supplemental content to
J Med Libr Assoc. Jan;109(1):dx.doi.org/10.5195/jmla.2021.1031
www.jmla.mlanet.org
© Schvaneveldt, Stellrecht 2021

Assessing the roles and challenges of librarians in dental systematic and scoping reviews

Nena Schvaneveldt, AHIP; Elizabeth M. Stellrecht

APPENDIX

Survey instrument

1. Have you been involved in a dental (oral health, dentistry, dental hygiene, etc.) specific systematic or scoping review?

- ☐ Yes
- ☐ No

2. Are you a librarian or information specialist?

- ☐ Yes
- ☐ No

Demographics

3. How many years have you been a librarian or information specialist?

- ☐ 0–5
- ☐ 6–10
- ☐ 11–20
- ☐ >20

4. What kind of institution do you work in?

- ☐ Academic
- ☐ Association
- ☐ Hospital/clinic
- ☐ Other (please specify)

5. Where are you from?

- ☐ United States
- ☐ Canada
- ☐ Other (please specify)

JMLA Journal of the Medical Library Association

SC
Supplemental content to
J Med Libr Assoc. Jan;109(1):dx.doi.org/10.5195/jmla.2021.1031
www.jmla.mlanet.org
© Schvaneveldt, Stellrecht 2021

Reviews involved in

6. How many non-dental systematic or scoping reviews have you been involved in, regardless of state of publication?

[text entry]

7. How many dental systematic or scoping reviews have you been involved in, whether they were published or not?

[text entry]

8. Of these dental systematic or scoping reviews, how many are still in progress (e.g., are in planning stages, searching, and/or data analysis is underway, etc.)?

[text entry]

9. How many dental *systematic reviews* have you been involved in that were published or accepted for publication?

[text entry]

10. How many dental *scoping review*s have you been involved in that were published or accepted for publication?

[text entry]

11. Of the dental *systematic or scoping* reviews you contributed to, how many were abandoned or never published?

[text entry]

12. How many dental systematic or scoping reviews have you collaborated on (i.e., working longer-term with research team, engaging in a wide array of duties including database selection, search strategy, defining/refining topic, possibly acknowledged, but not credited as coauthor)?

[text entry]

13. How many dental systematic or scoping reviews have you coauthored (i.e., worked in-depth with research team, developed strategy, written methods, credited with authorship)?

[text entry]

Roles and challenges

What roles have you taken on in a dental systematic or scoping review? [Spencer and Eldredge, 2018] Select all that apply.

14. Planning and protocol

- ☐ Clarifying what is involved in pursuing a systematic/scoping review
- ☐ Question formulation
- ☐ Guidance on selecting review methodology
- ☐ Searching for similar or previous reviews/protocols on topic
- ☐ Locating or recommending journals to publish manuscript
- ☐ Protocol development

JMLA Journal of the Medical Library Association

SC
Supplemental content to
J Med Libr Assoc. Jan;109(1):dx.doi.org/10.5195/jmla.2021.1031
www.jmla.mlanet.org
© Schvaneveldt, Stellrecht 2021

15. Information retrieval
- ☐ Selecting databases and sources to search
- ☐ Developing search strategy
- ☐ Searching grey literature
- ☐ Evaluation of search strategies

16. Managing results
- ☐ De-duplication of search results
- ☐ Documenting search results (e.g., for PRISMA diagram)
- ☐ Consulting and recommending the use of software and tools (e.g., Covidence, Rayyan, nVivo)
- ☐ Facilitating the use of systematic review software

17. Screening and data extraction
- ☐ Title/abstract screening
- ☐ Finding full text
- ☐ Full text screening
- ☐ Data extraction

18. Assessment
- ☐ Quality assessment
- ☐ Locating risk of bias tools
- ☐ Conducting risk of bias assessment

19. Manuscript preparation
- ☐ Writing methods section
- ☐ Writing sections other than the methods section
- ☐ Editing manuscript
- ☐ Citation management
- ☐ Other (please specify)

20. Which challenges have you encountered in conducting dental-specific systematic reviews? [Nicholson, McCrillis, and Williams, 2017]

Select all that apply (questions 21–23).

21. Challenges involving the librarian/information specialist
- ☐ I have inadequate training in systematic review methodology
- ☐ Lack of my own confidence in my ability to construct a systematic/scoping review search
- ☐ My lack of subject knowledge in dentistry
- ☐ My lack of time to conduct a review
- ☐ Lack of support from administration to develop my skills

JMLA Journal of the Medical Library Association

SC
Supplemental content to
J Med Libr Assoc. Jan;109(1):dx.doi.org/10.5195/jmla.2021.1031
www.jmla.mlanet.org
© Schvaneveldt, Stellrecht 2021

22. Challenges involving individual researcher/team lead
- ☐ Researcher has inadequate training in systematic review methodology
- ☐ Researcher is not tracking reasons for exclusion
- ☐ Researcher expects librarians to provide only administrative tasks
- ☐ Researcher is not using two-step screening process (i.e., first reviewing title/abstract then full article)
- ☐ The researcher does not follow a data extraction plan
- ☐ Researcher does not want to evaluate study quality as part of process
- ☐ Researcher does not have inclusion/exclusion criteria established at the beginning of process
- ☐ Researcher does not follow systematic review methodology
- ☐ Researcher is not using two screeners
- ☐ A student is leading the project, and the student's faculty mentor is not helpful
- ☐ Researcher refuses librarian credit for authorship

23. Challenges involving the entire team
- ☐ Lack of buy-in from other team members for librarian's role
- ☐ Research team's lack of time to conduct a review
- ☐ The research team is dysfunctional
- ☐ The research team cannot adhere to review's overall time table
- ☐ The research team cannot agree on question
- ☐ The research team has too many members
- ☐ Research team misunderstands amount of time to conduct systematic/scoping review
- ☐ Research team misunderstands rigor of systematic/scoping reviews
- ☐ The research team has too few members
- ☐ General lack of resources to conduct a review (e.g., access to e-resources, software, etc.)
- ☐ Research question is defined too broadly
- ☐ Question is defined too narrowly (i.e., search retrieves too few results to draw a conclusion)
- ☐ Other (please specify)

REFERENCES

1. Nicholson J, McCrillis A, Williams JD. Collaboration challenges in systematic reviews: a survey of health sciences librarians. J Med Libr Assoc. 2017 Oct;105(4):385–93. DOI: http://dx.doi.org/10.5195/jmla.2017.176.
2. Spencer AJ, Eldredge JD. Roles for librarians in systematic reviews: a scoping review. J Med Libr Assoc. 2018 Jan;106(1):46–56. DOI: http://dx.doi.org/10.5195/jmla.2018.82.
